# Chronic cough: A review and prospects

**DOI:** 10.1097/MD.0000000000045162

**Published:** 2025-10-10

**Authors:** Xiaoxuan Hu, Keli Zhang, Tao Liu, Xiao-ji Zhu

**Affiliations:** a Department of Pulmonary and Critical Care Medicine, Weifang No. 2 People’s Hospital, Weifang Respiratory Disease Hospital, Weifang, China; b Department of Respiration, The 80th Group Army Hospital of People’s Liberation Army, Weifang, China.

**Keywords:** chronic cough, diagnosis, epidemiology, etiology, pathogenesis, therapy

## Abstract

Chronic cough (CC) is a common respiratory disorder, with prevalence rates ranging from 10% to 20%. Its burden can be substantial, as patients often experience physical symptoms (e.g., stress urinary incontinence, sleep disturbance, chest pain), psychological distress (e.g., disappointment and depression), and social difficulties (e.g., social distress/isolation, reduced quality of life). Consequently, CC poses a frustrating challenge for both patients and clinicians. In this review, we summarize the current understanding of CC, including its epidemiology, pathogenesis, etiology, clinical features, auxiliary examination, diagnostic and differential diagnostic approaches, and treatment strategies, while also outlining future research directions. We highlight recent developments suggesting that the field is evolving, with new insights and practical tools for clinical researchers to improve the assessment and management of patients with CC.

## 1. Introduction

Cough is among the most frequent complaints among patients visiting respiratory clinics. Symptom duration classifies cough into acute (<3 weeks), subacute (3–8 weeks), and chronic (>8 weeks). This classification has crucial diagnostic implications, making thorough history-taking essential (Table 1).^[1]^ Chronic cough (CC) is prevalent in adults in developed countries (10–20%), with global rates varying from 3.9% to 30% depending on geography, demographics, and environmental exposures (e.g., air pollution, occupational hazards).^[2]^ For patients, CC can be distressing due to symptom severity, its complications, and lifestyle modifications required to avoid triggers. The impact extends beyond physical symptoms (e.g., stress incontinence, sleep disturbance, chest pain) to psychological (e.g., disappointment, depressive states) and social domains (e.g., social anxiety/isolation, decreased quality of life).^[3]^ The etiology of CC is complex and multifactorial, often involving cough variant asthma (CVA), gastroesophageal reflux cough (GERC), and upper airway cough syndrome (UACS). Some patients may progress to refractory chronic cough (RCC) or unexplained chronic cough (UCC), both significantly impair quality of life. CC also imposes a substantial societal burden through healthcare costs and productivity losses. Therefore, developing strategies to manage symptoms, mitigate underlying causes, and enhance timely and accurate diagnosis and treatment is critical for CC research.^[4]^

**Table 1:** **Classifications and etiologies of cough.** ^[1]^

Acute	Subacute	Chronic
• Upper respiratory tract infections	• Postinfectious	• Upper airway cough syndrome
• Lower respiratory tract infections	• Cough	• Gastroesophageal reflux
• Hay fever, allergic rhinitis	• Pertussis	• Asthma
• Inhalational exposure	• Angiotensin converting enzyme inhibitors	• Angiotensin-converting enzyme inhibitors • Chronic bronchitis • Tracheobronchomalacia • Bronchiectasis • Lung cancer

Reprinted from Ref. ^[1]^ doi: 10.1016/j.mcna.2020.08.013, which is licensed under a Creative Commons Attribution 4.0 International License.

Although international guidelines, such as GINA 2024 asthma recommendations, provide a framework for diagnosis and management, clinicians continue to encounter significant real-world challenges.^[5]^ For example, diagnostic heterogeneity persists: some studies use the outdated “>3 months” definition, which can underestimate prevalence by excluding protracted or subacute cases.^[6]^ CC has a complex and diverse etiology, with rare or unclear causes accounting for approximately 20% to 30% of cases, including conditions such as retrosternal goiter and central nervous system regulatory disorders.^[7]^ Treatment options also have limitations: for example, P2X3 receptor antagonists are effective but often cause dysgeusia, and RCC patients frequently respond poorly to conventional therapies.^[8]^

Recent advances include large-scale epidemiological data, such as Chinese national health insurance claims data from 9 major cities (e.g., Beijing, Shanghai, Guangzhou), showing an adult CC prevalence of 8.88%, with women representing 55.24% of cases. This gender disparity underscores the need for targeted investigations.^[9]^ New insights regarding CC pathogenesis reveal that cough hypersensitivity involves both peripheral sensory receptors (e.g., TRPV1 and TRPA1 receptors) and central nervous system structures (e.g., periaqueductal gray in the midbrain).^[10]^ Innovative treatment approaches have emerged, including non-pharmacological therapies such as deep diaphragmatic breathing and vagus nerve modulation, as well as targeted pharmacological agents (e.g., TRPV4 antagonists), which are now in clinical trials.^[11–13]^

However, future research and clinical efforts should focus on unifying epidemiological standards, adopting the 8-week criteria from international guidelines, and conducting large-scale multicenter studies; clarifying the neural mechanisms underlying CC through brain imaging to explore central regulatory pathways; optimizing treatment strategies by integrating traditional Chinese medicine (e.g., *Schisandra chinensis, Asarum sieboldii*) with personalized therapies. These remain pivotal directions for addressing clinical needs and driving future advancements.^[14–16]^

This article systematically reviews the epidemiological characteristics, pathogenesis, diagnostic approaches, and treatment options for CC, discusses key barriers in clinical implementation, and proposes strategic research directions to bridge these gaps, ultimately aiming to inform patient-centered prognostic improvement and evidence-based public health policies across multiple scales.

## 2. Historical development of the concept in CC

In 1876, Jean-Martin Charcot, a young Parisian physician, first described a cough accompanied by the loss of consciousness or an episode of coughing with laryngeal irritation at a meeting of the Societe de Biologie. Getchll further illustrated that the chief symptom is an attack of dizziness or the loss of consciousness preceded by a tickling sensation in the larynx and a usually severe cough. The symptoms were similar to those of the vertigo of Meniere’s disease, and, in laryngological literature, this condition is usually referred to as “laryngeal vertigo.” Whitty explained that the neurologist tended to regard the case as epileptic in origin and classified it under the heading of “laryngeal epilepsy” in 1943. The association of the larynx with epilepsy is most likely the result of 1 of 2 phenomena: either the local effect may be an aura of true cryptogenic epilepsy, or irritation in the larynx may be an appropriate stimulus causing an attack of reflex epilepsy. O’Doherty termed “tussive syncope” without or with convulsions cannot be fully explained by the present theories of hemodynamics and abnormal vascular reflexes. “Tussive syncope” would be more accurately classified as a symptomatic epileptic phenomenon; Kerr and Derber called the syndrome of “cough syncope,” which was characterized by sudden syncope following vigorous unproductive cough, it occurs in middle aged men who are robust and extroverted, and who indulged heavily in smoking, drinking, and eating in 1953. In the mid-19th century, typical cough syncope often occurred in middle aged men, obesity, smoking, and chronic obstructive pulmonary disease, affecting cardiopulmonary and cerebral vessels.^[17]^

Based on the traditional concept, CC is caused by an acute cough and is associated with respiratory viral infection. CC can be a frustratingly difficult problem for patients and clinicians. People with CC are distressed by the frequency and severity of the symptom, its complications, and how it causes them to modify their activities to avoid situations that might trigger cough. Progress in addressing these issues has been slow and intermittent.^[18]^ CRC or UCC has not been defined.^[19,20]^

In 2011, the European Respiratory Society expert group redefined CC as cough hypersensitivity syndrome (CHS), the main mechanism of which is the dysregulation of sensory neural pathways and central processing in the regulation of cough reflex (Table 2).^[21–23]^ CHS is an umbrella term or phenotype for a variety of complex processes and different types of coughs, such as CVA, GERC, post-nasal drip syndrome (PNDS), UACS, and non-asthmatic eosinophilic bronchitis (NAEB; Table 3).^[22,24]^ In addition, certain medications, such as angiotensin-converting enzyme inhibitors (ACEIs), can increase cough sensitivity.

**Table 2:** **Evidences for neuropathology in cough hypersensitivity.** ^[21]^

Category	Characteristics
• Clinical profile	• Cough triggered by trivial stimuli such as cold air, perfume, stress, exercise, singing, or talking (allotussia) • Urge-to-cough sensation • More coughs evoked by tussigen inhalation (hypertussia)
• Sensory neural activation in the airways	• Phenotypic switch of sensory neurons by respiratory virus infection, allergen, or air pollutant • Increased neuropeptides in bronchoalveolar lavage fluids • TRPVI upregulation in bronchial epithelial nerves • Decreased activation in brain areas implicated in cough suppression
• Central neural alterations in cough processing • Clinical trials	• Proven efficacy of drugs with neuromodulatory properties

TRPV1 = transient receptor potential vanilloid-1.

Reprinted from Ref. ^[21]^ doi: 10.4168/aair.2017.9.5.394, which is licensed under a Creative Commons Attribution 4.0 International License.

**Table 3:** **Causes of chronic cough and further investigations to consider.** ^[23]^

Disease process	Features	Investigations
• Cough variant asthma	• Airway hyperresponsiveness, no shortness of breath or wheezing	• Peak expiratory flow variability, exhaled nitric oxide, assessment of airway hyperresponsiveness
• Eosinophilic bronchitis	• Chronic cough without ether features of asthma	• Induced sputum/assessment of airway hyper-responsiveness/lung function tests
• Gastro-oesophageal reflux disease • Upper airway cough syndrome/sinus disease/external ear disease (mediated by Amold’s nerve)	• Dyspepsia/regurgitati on/obesity • Postnasal drip/nasal symptoms/facial pain or fullness/ear symptoms/atopy	• pH studies and manometry • Nasendoscopy/laryngosecopy/CT sinuses

CT = computed tomography; pH = potential of hydrogen.

Reprinted from Ref. ^[22]^ doi: 10.1136/dtb.2018.000014, which is licensed under a Creative Commons Attribution 4.0 International License.

**Figure 1.**
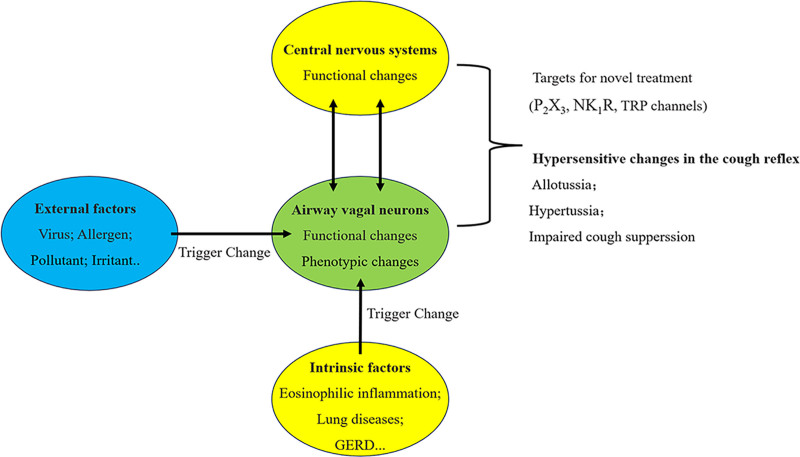
Schematic presentation of pathophysiologic components of cough hypersensitivity syndrome. Functional or phenotypic changes in peripheral sensory neurons are the first-level adaptive host responses in terms of developing cough reflex hypersensitivity, and these changes may be accompanied by functional changes in the central neural processing of the cough reflex. In fact, these changes are now considered central to chronic cough. Primarily, the changes may be triggered by external factors such as viral infection, allergens, air pollutants or irritants, as well as by intrinsic factors (or disease conditions) such as eosinophilic inflammation, lung diseases, or gastroesophageal reflux disease. Patients with chronic cough commonly present with hypersensitivity, including allotussia, hypertussia, and impaired cough suppression, although the pattern of hypersensitivity may differ among individuals. It is unknown how airway vagal sensory neurons interact with the central nervous system in developing and maintaining the chronic hypersensitive status of the cough reflex. Meanwhile, novel antitussives are being developed for patients with chronic refractory cough, targeting specific neuronal pathways implicated in the cough reflex, such as P2X3, neurokinin-1-receptor NK1R, or TRP channels. Reprinted from Ref. ^[1]^ doi: 10.3904/kjim.2020.013, which is licensed under a Creative Commons Attribution 4.0 International License. NK1R = neurokinin-1-receptor, P2X3 = P2X purinoceptor 3, TRP = transient receptor potential.

In recent years, cough induced by increased sensitivity of neural pathways has become a common clinical feature of CC.^[4]^ This new concept of cough hypersensitivity has changed the management strategy of CC, which is expected to relieve the pain associated with persistent cough.^[25,26]^ A total of 12 experts from the Italian National Institute of Pneumonia, National Institute of Health, Higher Institute of Health, Institute of Social Sciences, Italian Society of Allergy, American Clinical Society of Immunology, Italian Society of Geriatrics, Italian General Society of Medicine, and Italian Society of Pneumonia agreed that the central nervous system plays an important role for regulating cough on March 3, 2021. Increased central cough sensitivity is an important pathogenesis of RCC. Therefore, central sensitization may be an important factor in CC or CHS, as depicted in Figure 1.^[1]^

Over the past 2 decades, the CC concept has undergone a paradigm shift, transitioning from simplistic phenotypic characterization to molecular-level mechanistic dissection (e.g., neuroimmune mechanisms), and from uni-causal attribution to complex multisystem network interactions involving pulmonary, neurological, and immunological axes. The current key challenge lies in establishing multimodal cross-disciplinary phenotypic classification standards and facilitating personalized treatment through the integration of translational medicine approaches.

## 3. Epidemiology of CC

The global prevalence of CC is approximately10% in adults. Particularly, its prevalence in adults ranges from 10% to 20% in Europe, America, and Australia; 2% in Africa; and 18% in Oceania.^[27]^ In the general population of Copenhagen, Denmark, the prevalence of CC was 4% overall, with a prevalence of 3% in never-smokers, 4% in former smokers, and 8% in current smokers.^[28]^ Moreover, the prevalence of CC in a meta-analysis was 6.2% in adults in China.^[9]^ The incidence of CC ranges from 1.2 to 5.7 per 100 person-years in population-based studies of adults ≥45 years of age in Belgium and Canada.^[9,29]^ However, no global or continental-level data are available. The longitudinal epidemiology of CC and cough hypersensitivity is largely unknown, although cough may persist for longer than 5 years despite treatment in adults with CC.^[30–32]^ The prevalence of CC in children is not as clearly defined as in adults. Overall, the prevalence of CC in children ranges from 1.1% to 21.9%^[33–35]^; the difference in these estimates is possibly due to the differences in the method of data collection, definition of CC used, setting studied (such as high-income vs low-income country), and the age of children studied.

CC is more likely to occur in middle aged women; however, there are some regional variations. Among the 10,032 patients in the United Kingdom, the Netherlands, Sweden, South Korea, and the United States, most were of age 60 to 69 years; in China, most patients were of age 30 to 39 years.^[4]^ CC is generally more prevalent in women (66–73%) than in men, and the gender differences can mainly be related to the airways C-fibers; polymodal nonmyelinated endings capable of detecting chemical substances and temperature changes.^[36]^ The influence of gonadal sex hormones on ion channels has long been recognized in human physiology. Moreover, females are predominantly affected by functional disorders involving ion channels. Epidemiological data have shown that most patients attending specialized cough clinics worldwide are women.^[25]^

In the United Kingdom, only 0.2% of the 2,00,000 patients with a cough have a clear diagnosis of CC. In Italy, the prevalence of CC ranges from 14% to 18% and is steadily increasing.^[37]^ In 2018, a recent prospective survey of 11 cases/1000 people/yr, the prevalence of CC was 10% to 20% in 9824 people aged >45 years old in Western countries.^[38]^ In 2013, the total prevalence of CC was 10% and was significantly increasing in developed countries.^[35]^

CC can last for several years. A European cross-sectional survey of 1120 individuals revealed that 20% of the patients had a cough duration of at least 10 years.^[39]^ In a questionnaire survey conducted in the United Kingdom (n = 373), nearly 10% of the patients had a cough duration of <30 years.^[40]^ A Finnish study (n = 421) revealed that 57% of the patients had CC lasting for 1 year and 26% continued to have CC after 5 years.^[41]^ In a longitudinal analysis of 42 cough cases lasting from 7 to 10 years in the United Kingdom, 24% of the patients reported no change in severity, and 36% reported an increased severity over time.^[42]^ A retrospective analysis of 2,00,000 cough patients in the United Kingdom confirmed that 18% of the patients had at least 1 cough-related symptom and that only 0.2% had a definite diagnosis of CC.^[43]^

The observed differences in the CC prevalence reflect the complex interplay of environmental exposures, genetic susceptibilities, socioeconomic/behavioral factors, and healthcare-system access and efficacy. In clinical practice, diagnosis and treatment protocols should be tailored to regional characteristics (e.g., environmental exposures, healthcare infrastructure), whereas, at the public health level, systematic environmental interventions and research standardization efforts should be advocated to mitigate disease burden through targeted prevention and evidence-based policies.^[44,45]^ The transnational variations in CC stem from the interplay of underlying epidemiological factors (such as environmental exposures, etiological profiles, and demographic distributions) as well as methodological heterogeneities (such as diagnostic criteria variability, study-design diversity, and confounder control inconsistencies). Future research must therefore prioritize standardizing diagnostic criteria (e.g., adopting an 8-week duration threshold), optimizing sample representativeness through stratified sampling, and deepening investigations into environmental exposures and genetic predisposition mechanisms.^[46]^

## 4. Pathogenesis of CC

**Figure 2.**
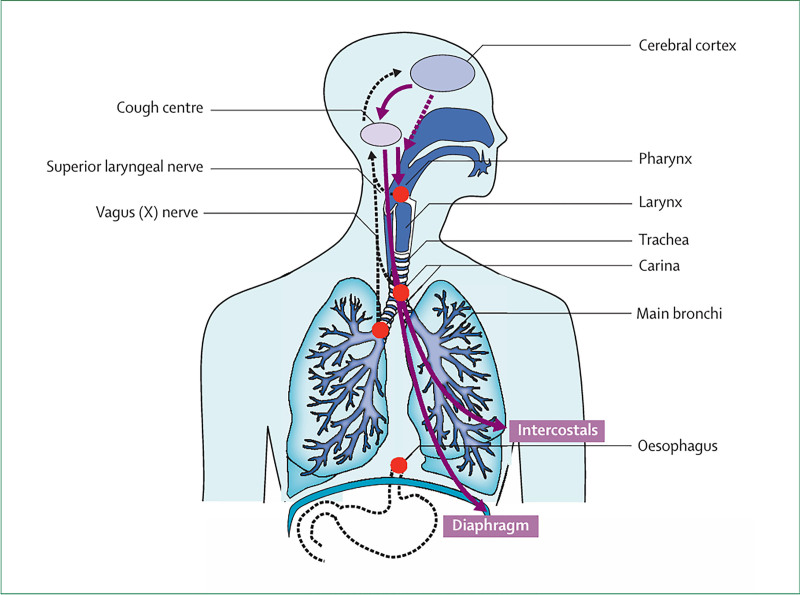
Anatomical representation of neural pathways for cough. Cough receptors (in red color) at the airway bifurcations, in the larynx, and at the distal esophagus are linked to cough afferents through the vagus and superior laryngeal nerves in the cough center and the cerebral cortex. Efferent pathways coordinate the muscle response that leads to a cough. Reprinted from Ref. ^[47]^ doi: 10.1016/S0140-6736(08)60595-4, which is licensed under a Creative Commons Attribution 4.0 International License.

Cough is an important protective reflex, and its physiological function is to clear secretions from the lower respiratory tract. The involuntary cough reflex is completed through a complete cough reflex arc, which consists of cough peripheral receptors, vagal afferent nerve, higher cough center, efferent nerve, and effectors (such as diaphragm, larynx, chest, and abdominal muscle groups). Extra-respiratory cough receptors (specific excited cells) are located in the neighboring organs, such as the pericardium, esophagus, diaphragm, stomach, ear canal, and eardrum (Fig. 2).^[26,48]^

The cough receptor is converted via mechanical or chemical stimulation into an electrical signal to initiate a reflex arc. Chemoreceptors exist outside the respiratory tract and are triggered by the actions of acids, cold, heat, capsaicin/capsaicin-like compounds, and fragrances. Mechanoreceptors exist in the throat, trachea, and bronchus branches and can be triggered by inhaled particles. Once stimulated, pulses arising from the cough receptors are transmitted through the vagus nerve to the medulla in the cough center. The cough center is controlled by the cerebral cortex and generates outgoing signals through the phrenic nerve, vagus nerve, and spinal motor nerve, which reach neurons distributed in the diaphragm, throat, trachea, and bronchus.

**Figure 3.**
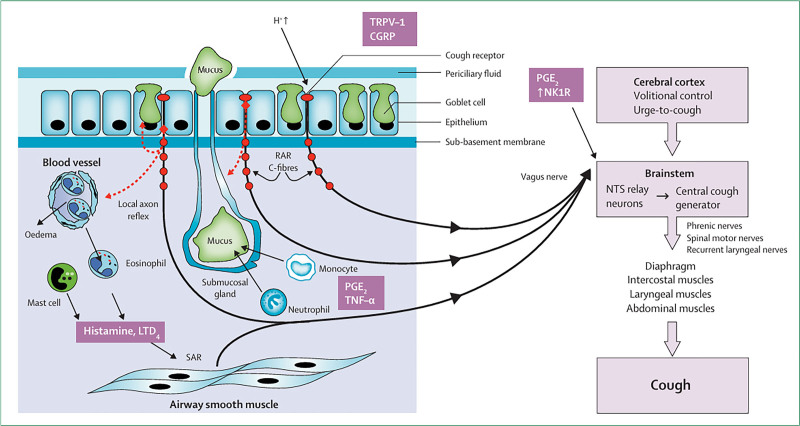
Representative scheme of afferent and efferent pathways regulating cough. The pathophysiology of the enhanced cough reflex involves laryngeal and pulmonary receptors, such as RARs, C-fibers, and SAFs, and cough receptors provide input to the brainstem medullary central cough generator through the intermediary relay neurons in NTS. The central cough generator then establishes and coordinates the output to the muscles that cause coughing. An output to the airway smooth muscles and mucosal glands (not shown) can be seen. The cerebral cortex can control the motor output of cough volitionally or influence the urge-to-cough sensation. Factors that act in the upper airways or brainstem to enhance the cough reflex are shown. Reprinted from Ref. ^[47]^ doi: 10.1016/S0140-6736(08)60595-4, which is licensed under a Creative Commons Attribution 4.0 International License. CGRP = calcitonin gene-related peptide, LTD4 = leukotriene D4, NK1 = neulokinin-1, NTS = nucleus tractus solitarius, PGE2 = prostaglandin E, RARs = rapidly adapting receptors, SAFs = slowly adapting fibers, TRPV = transient receptor potential vanilloid, TNF = tumor necrosis factor.

The coordinated actions of the expiratory muscle and the pelvic sphincter muscle produce a violent gas that serves as a driving force to ensure gas exchange in the lungs.^[47]^ The activation of C-fibers in the lungs can inhibit coughing in anesthetized animals. The activation of extrapulmonary C-fibers and rapidly adapting receptors (RARs) can cause coughing. Anesthetized guinea pigs have a myelin afferent nerve subtype associated with pulmonary RARs and slowly adapting receptors (SARs), which play an important role in inducing cough (Fig. 3).^[48,49]^

In RARs and C-fiber receptors, a transient receptor potential vanilloid-1 (TRPV-1) channel exists that can be activated by heat, acid, bradykinin, arachidonic acid derivatives, and adenosine triphosphate. The TRPV-1 expression is increased in patients with CC.^[50,51]^ In sensitized guinea pig models, TRPV-1 inhibitors suppressed tissue responses resulting from allergen attack. Bradykinin and prostaglandin E2 and F2α can enhance the responsiveness of capsaicin to coughing by controlling specific sodium channels.^[52,53]^ RARs are sensitive to cigarette smoke, acidic and alkaline solutions, hypotonic and hypertonic saline, mechanical, pulmonary congestion, atelectasis, bronchoconstriction, and reduced lung compliance. In addition, C-fiber receptors are highly sensitive to chemicals, such as bradykinin (inflammatory mediators), capsaicin (chili vanilla extract), and hydrogen ions (acidic pH), all of which can enhance coughing.^[54,55]^ Thus, the pathogenesis of cough may involve eosinophilic inflammation and airway hypersensitivity.

In summary, cough reflex regulation is coordinated by multiple receptors and neural pathways. TRPV1/C-fiber mediates chemosensitivity and contributes to CC hypersensitivity. RARs respond to mechanical stimuli, promptly initiating defensive coughing. SARs may modulate cough threshold through stretch-dependent modulation. Afferent nerve subtypes interact via complex mechanisms. For example, C-fibers indirectly activate RARs through neuropeptide release, whereas inflammatory mediators (e.g., histamine) enhance RARs’ mechanical sensitivity. In addition, TRPV1 and TRPA1 exhibit structural and functional synergy, as demonstrated by the requirement for dual blockade (e.g., TRPV1 + TRPA1 inhibitors) toward suppressing bradykinin-induced coughing. Capsaicin-sensitive vagal neurons have emerged as the key targets of the cough-specific neural circuit. These findings establish a robust theoretical basis for the development of precision antitussive therapies, including P2X3 antagonists and selective TRP channel inhibitors (e.g., TRPV1/TRPA1 dual-blockers), thereby paving the way for targeted CC treatments.^[56,57]^

The key pathophysiological roles of TRPV1 in CC are as follows: peripheral sensitization: TRPV1, primarily expressed at the terminals of vagal C-fibers, is a polymodal nociceptor. It is activated by capsaicin, acidic environments (pH ≤ 5.9), and inflammatory mediators (e.g., bradykinin and prostaglandin E2), resulting in Ca²^+^ influx and generation of action potential. These signals are then transmitted to the nucleus tractus solitarius in the medulla oblongata, initiating the cough reflex. Synergistic interaction with TRPA1: both TRPV1 and TRPA1 share components of the PLC/IP3-Ca²^+^-signaling cascade, which activates protein kinase C. Upon activation by inflammatory mediators, they cause sustained Ca²^+^ influx and channel potentiation, forming a positive feedback loop that amplifies airway hyperresponsiveness. TRPA1 is more sensitive to cold stimuli (e.g., cold air) and oxidative stressors (e.g., cinnamaldehyde), whereas TRPV1 responds to heat and acid. Amplification of neurogenic inflammation: vascular and inflammatory effects: Neuropetides (e.g., substance P, calcitonin gene-related peptide) dilate blood vessels, increase permeability, and recruit inflammatory cells, creating an “inflammation-cough” vicious cycle. Sensitization of peripheral mechanoreceptors: neuropeptides released by C fibers enhance the mechanical sensitivity of RARs, triggering coughing by minimal stimuli. Central sensitization facilitation: plasticity changes: the prolonged activation of peripheral TRPV1 leads to central synaptic plasticity alterations (e.g., *N*-Methyl-D-aspartate receptor upregulation), which lowers the activation threshold of the cough center and manifests as CHS. Indirect activation of P2X3: TRPV1 indirectly activates purinergic receptor P2X3 by regulating ATP release, which plays a crucial role in RCC.^[58,59]^

## 5. Etiological structure of CC

### 5.1. Cough associated with CVA

CVA with cough as the only or the main symptom, accompanied by airway hyperresponsiveness, accounts for 10% to 40% of all CC cases.^[55,60]^ Cough is mostly irritant and dry cough: it is light during the daytime, but serious at nighttime. Airway hyperresponsiveness is present, but there is no shortness of breath or wheezing, and anti-asthmatic drugs are effective in treating CVA.^[24]^ Epidemiological studies have revealed that CVA causes 32% to 34% of all CC cases in China. In Europe and the US, 25% to 32.6% of all CC patients and 30% to 35.7% of all CVA patients eventually develop classic asthma.^[56]^ Thus, the US and European guidelines emphasize the value of bronchial provocation testing in diagnosing CVA. More than half of the CVA patients can develop small airway dysfunction, which continues to persist after short-term anti-asthmatic treatment; in fact, it is a unique clinical and pathophysiological feature of CVA.^[61]^ Based on the sputum cytology, the types of inflammation in asthmatic patients can be categorized into 4 phenotypes: eosinophilic, neutrophil, mixed cell, and oligocytic. Eosinophil and neutrophil phenotypes are common in CVA; however, oligogranulocyte phenotypes are easier to manage clinically.^[23]^

### 5.2. Cough associated with GERC

The clinical manifestations of GERC include heartburn, persistent cough, asthma, sore throat, laryngitis, and/or hoarseness in extra-esophageal nonspecific symptoms, while coughing is the only symptom in 75% of all GERC patients. Neuroimmune changes in the airway and esophagus, sensitization of the peripheral and central nervous systems, increased sensitivity of the cough reflex, and hypersensitivity of esophageal viscera contribute to coughing due to gastroesophageal reflux. The incidence of GERC was 2.5% in China, <10% of that in Asia, 27.8% of that in North America, 25.9% of that in Europe, and 51.2% of that in Greece.^[27,41]^ In China, GERC accounted for 40.7% of CC cases in 2016. The 24-hour esophageal pH-monitoring technique is the gold standard for GERC diagnosis. Modern electronic instruments can measure the internal pressure of the esophagus and the fluidity-related dynamic parameters of the esophageal contents. Approximately 60% of all GERC cases can be effectively treated with proton pump inhibitors (PPIs); however, the short-term use of PPIs is associated with a 1.5-fold increase in the risk of community-acquired pneumonia.^[9,62,63]^ In fact, in a past study, after inhaling capsaicin for 2 to 5 times, the number of patients with irritant cough induced by GERC became significantly lower than that with irritant cough induced by UACS.^[29]^

### 5.3. Cough associated with NAEB

NAEB is a common cause of CC and is characterized by persistent coughing and airway eosinophilia, similar to the pathological changes in asthma but without airway hyperreactivity and higher levels of mast cells, histamine, and prostaglandin D2 in the sputum.^[64]^ NAEB accounts for 10% to 30% of all CC cases.^[1]^ Differences in the site of airway inflammation, the level of inflammatory mediators, imbalance in the metabolic pathways, and the degree of airway remodeling determine the underlying pathogenesis of NAEB and typical asthma.^[43]^ The latest Chinese guidelines on cough diagnosis and treatment suggest that an increase in the number of sputum eosinophils (>2.5%) is an important diagnostic criterion. In a study of 234 patients with NAEB followed up for 1 year, eosinophils were detected in the sputum of 30% of the patients, whereas 5.7% of the patients developed mild asthma without any progressive decline in their lung capacities.^[49]^

### 5.4. Cough associated with UACS

UACS is characterized by CC and is accompanied by upper respiratory tract symptoms, including episodic or persistent cough, abnormal nasopharyngeal secretion, or frequent throat clearing. Its common concomitant manifestations include nasal congestion and a runny nose.^[47]^ In 2006, the American College of Chest Physicians defined PNDS as UACS. UACS is the most common cause of CC in the United States. In China, UACS accounts for 17% to 22% of all CC cases, whereas in Europe, it accounts for 6% to 21%. The pathogenesis of UACS includes the early theory of postnasal drip, the theory of chronic airway inflammation, and the recent theory of sensory nerve hypersensitivity.^[65]^ A cohort study in Japan reported that 146 of 230 patients with CC were diagnosed with UACS, which is considered to be the main cause of CC in Japan.^[66]^

The common causes of CC include CVA, GERC, NAEB, and UACS (contributing to 90%).^[55]^ Rare causes include obstructive sleep apnea syndrome, tracheobronchomalacia, persistent bacterial bronchitis, diffuse panbronchiolitis, tracheobronchial tuberculosis, *Mycobacterium tuberculosis*, mediastinum tumor, arrhythmia, connective tissue diseases (such as rheumatoid arthritis, systemic lupus erythematosus, Sjogren’s syndrome), inflammatory bowel disease (such as ulcerative colitis and Crowe’s disease), somatic cough syndrome, giant cell arteritis, recurrent polychondritis, idiopathic pulmonary fibrosis (IPF), and chronic laryngeal cough. In addition, the following drugs induce CC: ACEI, angiotensin receptor blockers, sitagliptin, calcium channel blockers, fentanyl, latanprost, topiramate, phenytoin sodium, methotrexate, molinyl ethyl, and omeprazole.^[67]^

The etiological structure of CC primarily involves noninfectious airway inflammation (e.g., CVA and NAEB) and UACS, with postinfection persistence, gastroesophageal reflux, and environmental exposures as the key synergistic factors. In children, special attention should be paid to respiratory infections and foreign body inhalation, whereas in adults, asthma and GERC should be screened as common etiologies. Confirming the cause of CC requires a multimodal approach that combines medical history, targeted provocation tests (e.g., bronchial challenge tests), and chest imaging studies so as to differentiate nonbacterial etiologies. Considering that most cases are noninfectious, avoiding antibiotic overuse is critical. Thus, timely referral to a specialist for comprehensive evaluation is essential to improve patient outcomes.

## 6. Clinical manifestations and auxiliary examination of CC

Cough is a protective reflex that prevents aspiration. However, in some patients, it becomes persistent and distressing, often evolving into CC, defined as a cough lasting 8 weeks in adults and 4 weeks in children, in association with many pulmonary and extrapulmonary diseases.^[4]^ In adults, CC is sometimes accompanied by low-grade fever and mechanical or chemical airway irritation. In the United Kingdom, 10% to 15% of CC patients seen in respiratory clinics show airway submucosal pathology similar to asthma, while NAEB involves eosinophilic infiltration confined to the central airway mucosa, with accompanying T lymphocyte and mast cell infiltration.^[63,68]^ CC is frequently associated with CVA, GERC, NAEB, and UACS; however, in some patients, the cough remains extremely difficult to control. Symptoms of CC can persist for years, requiring repeated clinic visits, long-term empirical therapy, and frequent diagnostic testing.^[3]^

In elderly adults, cough-related complications are more common, with urinary incontinence being frequent. Aspiration pneumonia can be fatal in those with weakened cough reflexes due to dementia, stroke, or Parkinson’s disease.^[69]^ UACS, CVA, and GERC together account for 85% of CC cases in elderly people.^[44]^ In respiratory clinics, 38% of patients present with cough as their main symptom, and in 10%, it is the only symptom; of these, UCC accounted for 46% of CC patients.^[70]^

Chest auscultation may reveal wheezes and prolonged expiration. Coarse crackles can indicate bronchiectasis, whereas fine, late inspiratory crackles suggest diffuse parenchymal lung disease.^[19]^ Chest radiographs and pulmonary function tests are recommended for all patients suspected of CC. Additional tests include exhaled nitric oxide, chest CT or MRI, or FDG-PET-CT, gastroscopy, polysomnography, and bronchoscopy.^[26,71]^ In adults, airway hyperresponsiveness testing, NAEB evaluation, or glucocorticoid therapy is advised.^[72]^ An ear, nose, and throat examination may reveal nasal obstruction from inflamed turbinates or polyps, and indirect laryngoscopy may show laryngeal/pharyngeal irritation suggestive of proximal gastroesophageal reflux.^[19]^ Among 253 patients analyzed by Nguyen et al,^[73]^ the visual analog scale was found to be a simple, noninvasive, and effective tool for assessing cough severity in clinical settings.

## 7. Diagnosis and differential diagnosis of CC

A detailed history should document cough type (wet vs dry), triggers (such as conversations, meals, perfumes, smoke, fumes, or chemicals), and characteristic features (e.g., throat clearing) of patients with suspected CC to determine its cause. Physical examination may reveal signs of obstructive lung disease, lung cancer, bronchiectasis, pulmonary fibrosis, or cardiac failure, although findings are often nonspecific. Likewise, no features easily distinguish CVA. Asking patients to inhale sometimes provokes paroxysmal coughing.^[19]^ Assessment should also address severity, frequency, intensity, triggers, and impact on quality of life.^[26]^

UCC is a serious disease that occurs in 5% to 10% of patients and often requires referral to a cough specialist clinic (46% of cases [n = 127] in 1 cohort).^[10]^ UCC patients experience a severe decline in quality of life. Despite systematic investigation and treatment, patients with CC experience cough for months or years. UCC is diagnosed after systematic assessment and treatment for known causes of cough are completed. This analysis identified that various terms and descriptions are used to identify patients with UCC.^[74]^ Misono et al^[75]^ suggested that UCC in adult patients can be diagnosed as a cough that persists longer than 8 weeks and remains unexplained after investigation and supervised therapeutic trial(s) conducted according to published best practice guidelines.

RCC refers to a cough persisting despite optimal treatment of common and uncommon diseases with presumed correlation according to clinical guidelines.^[3]^ Both UCC and RCC may be manifestations of CHS, in which neural pathways are hyperresponsive rather than truly hypersensitive.^[19]^ CVA and NAEB are related to allergies, and allergic diseases increase CC risk more than threefold.^[27]^ CC affects more than half of IPF patients, with their CC arising from GERD, UACS, or IPF itself.^[22]^

In a group of studies evaluating 266 patients with CC, some were diagnosed as having asthma (29%), GERD (22%), and ACEI use (14%).^[76]^ A group of observational studies found that CC patients visiting cough specialist clinics had asthma (6–36%), GERD (0–41%), and rhinitis (8–56%).^[19]^ Chronic laryngeal cough is often associated with dysphagia, dysphonia, dyspnea, wheeze, or paradoxical vocal cord movements.^[77]^ Bonvini et al^[78]^ reported UCC in UCC in 57%, RCC in 27%, and other coughs in 16% of 100 patients with recurrent hard-to-control CC (69% were women, median duration of 3 years, and mean age of 58 years). These data suggest a significant rise in the prevalence of UCC and RCC, and the accompanying medical costs. Thus, these conditions have become health concerns with considerable social burden.

The key recommendations of official guidelines on CC from American College of Chest Physicians, European Respiratory Society, and British Thoracic Society are as follows: American College of Chest Physicians Guidelines: CC in adults is defined as a cough lasting ≥8 weeks with an unknown cause following routine examinations (e.g., chest x-ray, pulmonary function tests); assess airway hyperresponsiveness (e.g., bronchial provocation test) and sputum eosinophil count to determine airway inflammation; trial 2 to 4 weeks of inhaled corticosteroids (ICS) for adult patients, but if ineffective, discontinue; consider low-dose sustained-release morphine for managing RCC (e.g., starting at 5–10 mg bid) after careful risk–benefit assessment (e.g., addiction risk, respiratory depression); and avoid PPIs in patients without evidence of GERC.^[79]^ European Respiratory Society Guidelines (2020 edition): CC is defined as a cough lasting ≥8 weeks in adults and ≥4 weeks in children; global prevalence is approximately 10% in adults, with higher rates in women; emphasizes the role of CHS; classifies phenotypes (e.g., asthmatic, reflux-related, PNDS) to guide management; avoid CT in patients with normal chest x-ray and no physical signs (due to radiation risk and unclear diagnostic value); short-term trials of ICS (2–4 weeks) may benefit persistent CC in adults and children, but response varies; fractional exhaled nitric oxide (FeNO) and blood eosinophil counts have limited predictive value for the ICS response; and cough control therapy (e.g., speech therapy) can improve quality of life in RCC.^[80]^ British Thoracic Society Guidelines: Smoking cessation is required for smokers; treat eosinophilic inflammation (e.g., FeNO > 25 ppb) with ICS; stop ACEI medications after assessing the risk–benefit ratio. Combine FeNO (airway inflammation) and eCO (smoking exposure) for precision treatment guidance; chronic wet cough in children can be tentatively treated with antibiotics (e.g., amoxicillin–clavulanate); adults should use antibiotics cautiously and in specific scenarios (e.g., refractory chronic bronchitis with suspected infection or acute exacerbations); and macrolides (e.g., azithromycin) are indicated for specific populations (e.g., bronchiectasis, asthma with frequent exacerbations), and antibiotic resistance must be carefully monitored.^[81]^

In addition, CC should be differentiated from asthma, chronic bronchitis, chronic postnasal drip syndrome, chronic pulmonary interstitial fibrosis, emphysema, sarcoidosis, broncholung cancer, alveolar cell carcinoma, benign airway tumor, mediastinal tumor, foreign body aspiration, external auditory canal irritation, left ventricular failure, pulmonary infarction, aortic aneurysm, reflux esophagitis, recurrent aspiration, post-endobronchial suture cough, and ACEI-related cough (Table 4).^[21,24]^

**Table 4:** **Relevant conditions in the differential diagnosis of chronic cough.** ^[24]^

Frequency	Bronchopulmonary causes	Extrapulmonary causes
• Common	• Chronic (obstructive) bronchitis • Cough variant asthma • Bronchial asthma	• Upper airway cough syndrome • Gastroesophagageal reflux disease
• Uncommon	• Bronchial/lung cancer (primary and secondary) • Pertussis • Eosinophilic bronchitis	• Drugs (especially ACE inhibitors) • Cough of unexplained origin • Chronic left heart failure • Vocal cord dysfunction
• Rare	• Infections: tuberculosis, opportunistic (immunosuppression) • Tracheobronchial collapse • Bronchiectasis • Interstitial lung diseases • Cystic fibrosis	• Hodgkin’s lymphoma • Esophageal diverticulum

ACE = angiotensin converting enzyme.

Reprinted from Ref. ^[24]^ doi: 10.3238/arztebl.m2021.0396, which is licensed under a Creative Commons Attribution 4.0 International License.

Diagnosis and differentiation of common causes of CC: CVA, NAEB, and allergic cough respond well to glucocorticoid therapy (also termed hormone-sensitive cough). They are often clinically characterized by a nocturnal cough with chest congestion or wheezing. Symptoms of UACS are typically worse during the day and milder during the night. GERC is characterized by coughing upon just lying down or bending, often accompanied by acid reflux, heartburn, or indigestion. A wet cough is more common in infectious cough, with chronic bronchitis typically producing white mucus sputum in winter and spring. Hemoptysis should be noted to exclude the possibility of bronchiectasis, tuberculosis, or lung cancer, and must be differentiated from hematemesis and nasopharyngeal bleeding.

The diagnostic principles for CC are as follows: start with common causes: Assess for major etiologies (e.g., CVA, GERC, NAEB, UACS) and other frequently encountered conditions. Progress to rare diseases: If initial evaluations are negative, investigate less common conditions. Dynamic adjustment: Refine the diagnosis based on therapeutic response (e.g., reassess if symptoms improve or worsen with targeted therapies). Specialist referral for further evaluation: If standard treatment fails or alarming signs occur (e.g., hemoptysis, unexplained weight loss), refer to specialized departments (e.g., pulmonology, cardiology) for advanced tests (e.g., bronchoscopy, cardiac evaluation).^[82]^

## 8. Principles and evidence-based value of CC treatment

### 8.1. Evidence-based therapeutic principles

#### 8.1.1. CC etiological treatment

Etiological treatment strategies for CC vary by underlying cause. Antibiotics (e.g., amoxicillin, roxithromycin) are required to control bacterial pathogens causing infections such as chronic bronchitis or pneumonia. Combination therapy with inhaled glucocorticoids (e.g., budesonide) and short-acting bronchodilators (e.g., salbutamol) is recommended for CVA. GERC management involves treatment with acid-suppressing agents (e.g., omeprazole) and prokinetic agents. UACS requires targeted treatment of nasopharyngeal disorders, such as sinusitis or rhinitis.^[83]^

#### 8.1.2. CC drug treatment

Drug treatment for cough management: central-acting agents (e.g., dextromethorphan) or peripheral-acting agents (e.g., benproperine) are indicated for dry cough to suppress reflex activity; agents such as ambroxol and acetylcysteine are used to reduce sputum viscosity in productive cough; and traditional Chinese Medicine formulations, such as Su Huang Zhike Capsules, are used for cold-induced cough, and Feilike Mixture targets heat-induced cough. Traditional Chinese Medicine selection should follow individualized syndrome differentiation.^[84]^

#### 8.1.3. CC non-pharmacological treatment

Non-pharmacological treatment strategies: quitting smoking, avoiding irritants (e.g., spicy or acidic foods, cold beverages), and maintaining adequate hydration (≥1.5 L/d) can keep the throat moist; moderate-intensity aerobic exercises (e.g., swimming, Tai Chi) improve lung function, and respiratory training (e.g., pursed-lip breathing, diaphragmatic breathing) enhance cough control; and techniques such as ultrasonic nebulization and postural drainage, performed under professional supervision, aid in sputum clearance. All interventions should be individualized based on patient tolerance and comorbidities.^[85]^

#### 8.1.4. CC traditional Chinese medicine

Traditional Chinese medicine and complementary therapies: acupuncture targets specific meridian points to regulate qi and blood circulation, while massage techniques (e.g., Tui Na) relieve muscle tension and improve thoracic circulation, with both aiding in cough relief; herbal formulas (e.g., modified versions of Bu Fei Tang for lung qi deficiency) and dietary adjustments (e.g., warm, nonirritating foods) are tailored to individual conditions (e.g., cold or heat patterns) to address underlying imbalances.^[86]^

#### 8.1.5. CC psychological support and immunomodulation

Psychological support and immunomodulation strategies: CC can cause anxiety and sleep disturbances. Psychological interventions (e.g., cognitive behavioral therapy [CBT]) help improve patients’ quality of life and coping strategies; under medical supervision, immunomodulators such as Bacillus Calmette-Guérin polysaccharide nucleic acid (BCG-PSN) may be considered for patients with recurrent respiratory infections to enhance immune response. Allergy-related CC may require additional allergen-specific management.^[87]^

### 8.2. Evidence-based therapeutic value

#### 8.2.1. CC targeted treatments

Targeted treatments with antitussives and expectorants can reduce cough frequency and intensity, improving sleep and daily activities.

#### 8.2.2. CC targeted interventions

Targeted interventions addressing underlying causes (e.g., optimizing asthma management with inhalers or addressing GERC with PPIs and lifestyle modifications) can reduce the risks of complications such as pneumothorax and rib fractures secondary to prolonged coughing. Comprehensive evaluation by a multidisciplinary team ensures personalized strategies to halt disease progression.

#### 8.2.3. CC etiology establishment

Establishing an accurate etiology reduces misdiagnosis and overtreatment. When the underlying cause is identified, unnecessary use of antibiotics for noninfectious coughs and prolonged dependence on cough suppressants (which may mask the condition) should be avoided.

#### 8.2.4. CC individulized and long-term management

Individualized and long-term management requires tailoring plans to patients’ ages and underlying comorbidities. For example, elderly patients should receive preventive vaccinations to reduce the recurrence of respiratory infections.

In summary, CC management requires multidisciplinary collaboration, from etiological investigation to comprehensive intervention, combining pharmacological and non-pharmacological approaches. The goals are precise diagnosis, symptom control, and long-term complication prevention, with patient adherence and lifestyle modification essential for optimal therapeutic outcomes and minimizing recurrence risk.^[88]^

### 8.3. Advances in CC treatment

With the recent progress in medical technology and in-depth research on CC pathogenesis, treatment strategies for CC have gradually shifted from traditional empirical medication to etiology-driven, precise intervention. Many new diagnostic and treatment methods have emerged, including CC etiology classification, drug treatment (such as new P2X3 receptor antagonists and biological agents), non-pharmacological therapies (speech therapy and lifestyle modifications), personalized treatment, and special treatment considerations for elderly and pediatric CC patients. These advancements offer evidence-based frameworks guiding clinicians in personalized treatment decisions and empowering patients to actively manage triggers and adherence, which ultimately improves outcomes in CC care. CVA, UACS, BAEB, and GERC account for 70% to 95% of CC cases globally, with NAEB prevalence ranging from 5% to 33% (17.3% in large-scale Chinese epidemiological studies).^[9]^ At the 2025 International Aerosol Medicine Conference, experts proposed reconceptualizing RCC as a neurosensory disorder characterized by abnormalities in peripheral nerve signaling and central nervous system hypersensitivity, manifested as heightened cough reflexes and impaired central inhibitory control. This framework supports the development of novel targeted medications. AI-assisted diagnostic tools, which leverage machine learning to analyze cough sound characteristics and integrate clinical data, have been applied in CC to improve etiology identification accuracy. They show promise for widespread adoption in primary care practice.

#### 8.3.1. Latest breakthroughs in pharmacological treatment for CC

##### 8.3.1.1. Novel drugs targeting the cough reflex

P2X3 receptor antagonists reduce cough reflex hypersensitivity by blocking ATP-activated P2X3 receptors. Gefapixant, the first approved P2X3 receptor antagonist, has been authorized for RCC treatment of RCC in the European Union, United Kingdom, Japan, and Switzerland.^[89]^ Clinical trials have demonstrated a significant reduction in both cough frequency and severity, particularly in patients with CC who are unresponsive to other therapies.^[90]^ Camipixant, a novel P2X3 receptor antagonist currently in phase 3 trials, shows improved tolerability and a lower incidence of adverse events, including dysgeusia, based on initial data. These drugs offer promising options for refractory cases, particularly those with GERC or idiopathic CC unrelated to acid reflux.

Neuromodulators such as gabapentin and amitriptyline have shown potential value in RCC treatment. According to randomized controlled trials, they can significantly reduce cough frequency. They reduce cough sensitivity by modulating central nervous system excitability. While not formally approved for CC indications, off-label use has begun in clinical practice, particularly for patients with substantial psychological comorbidities or severe nocturnal coughing. Moreover, their use has been supported by small-scale trials showing efficacy in patients with RCC and comorbid anxiety.^[91]^

##### 8.3.1.2. Optimization strategies for anti-inflammatory treatment

ICS remains the first-line treatment for CVA, while short-course oral corticosteroids are preferred for NAEB induction. For NAEB patients, following short-course oral corticosteroids induction, maintenance with ICS, namely budesonide (e.g., 400 μg/d) or fluticasone propionate (e.g., 250–500 μg/d), for 3 to 6 months may help reduce recurrence. ICS alone is not the first-line treatment for NAEB. The optimal regimen should be individualized based on patient response. Randomized trials have demonstrated that ICS treatment with budesonide (400 μg/d) led to a reduction in recurrence rates from 41.9% at 1 month, 20.0% at 2 months, and 10.7% at 4 months (n = 200; for 1-month vs 2/4-month). These findings suggest that efficacy plateaus occur after 2 months, with the most marked reduction occurring within the first 2 months, rather than a strictly linear dose–response relationship.^[92]^ Sputum eosinophil counts of >3% at treatment completion predict significantly increased recurrence risk (e.g., a 2-fold higher risk in some studies). Therefore, ICS treatment duration must be individualized by serially monitoring sputum eosinophil levels (e.g., every 3–6 months posttreatment) to guide therapy adjustment.

Leukotriene receptor antagonists (e.g., montelukast) may serve as adjunctive therapy in select CC cases, such as CVA patients with comorbid allergic rhinitis (to target nasal symptoms) or NAEB patients with residual cough following optimal ICS/oral corticosteroids treatment. However, their role remains limited by low-quality evidence and potential side effects, and they are not first-line options.^[93]^ According to low-quality evidence from observational studies, in CVA patients with comorbid allergic rhinitis, montelukast (10 mg/day) may provide short-term symptomatic relief, comparable to high-dose ICS (e.g., cough severity reduction: 30% vs 40%). However, its anti-inflammatory effects against eosinophilic airway inflammation are limited and should not be used to replace ICS in patients with NAEB or eosinophilic bronchitis. Moreover, it must be used with caution because of potential neuropsychiatric side effects. Based on moderate-quality evidence, their use in concurrent UACS primarily targets upper airway inflammation (e.g., reducing nasal eosinophils, postnasal drip). Any potential benefit in the lower airways likely results from indirect mitigation of reflux stimuli rather than direct anti-inflammatory effects, with limited evidence supporting this secondary role. Leukotriene receptor antagonists should be reserved as adjunctive therapy, and not as first-line treatment, for UACS patients with comorbid CHSs (e.g., CVA).^[56]^

Biological agents may hold potential for refractory eosinophilic CC (e.g., targeting IL-5 in patients with sputum eosinophilia >3%), but phase III trials have lacked clinical significance.^[94]^ Their role remains unproven, and their use is restricted to clinical trials, given high costs, safety concerns, and the absence of endorsing guidelines. Anti-IL-5/IL-5R monoclonal antibodies (such as mepolizumab and reslizumab) are approved for severe eosinophilic asthma in patients with elevated blood eosinophils and recurrent exacerbations. The role of these monoclonal antibodies in NAEB has been investigated in small case series and observational studies, with limited evidence suggesting modest efficacy (cough reduction by 30–50%) and unproven long-term benefits. These agents also remain experimental for NAEB treatment, and their use is restricted to clinical trials. These drugs can significantly reduce airway eosinophilic inflammation levels, but only limited evidence from non-randomized studies supports their clinical benefit for patients with severe NAEB or those with overlapping asthma characteristics. While these drugs are potentially beneficial, treatment response to them is heterogeneous, with modest-to-variable improvements noted in cough symptoms and lung function. Larger RCTs are needed to confirm the magnitude of benefit and optimal patient selection criteria.^[95]^ Other targeted biological agents, such as anti-thymic stromal lymphopoietin (phase II trials ongoing), were promising for Th2-high airway diseases in preclinical trials, but their efficacy for recurrent NAEB remains unverified. Meanwhile, anti-IgE and anti-Siglec-8 agents have shown limited or no benefits in cough-related disorders, with the latter discontinued for safety concerns.^[96]^ Future options depend on trials targeting validated biomarkers (e.g., periostin, IL-33) in well-defined NAEB subgroups.

##### 8.3.1.3. Optimization of pharmacological treatment for GERC

The GERC management strategy continues to evolve. While PPIs (e.g., omeprazole) remain the first-line therapy for GERC, typically for 8 to 12 weeks, adding gastrointestinal motility drugs (e.g., domperidone) may benefit patients with comorbid delayed gastric emptying. However, this is supported by limited data from small trials and is associated with cardiovascular safety risks. For now, routine combination therapy is not recommended outside specialized settings. Baclofen, a GABA-B receptor agonist, exhibited limited efficacy in acid reflux reduction (18% decrease) by increasing lower esophageal sphincter pressure. However, its 30% central nervous system side effect rate (dizziness/sedation) and Food and Drug Administration black-box black-box warning restrict its use to only specific patients following strict risk-benefit assessment.^[92]^

##### 8.3.1.4. Systemic corticosteroids and novel antitussive drugs

For acute NAEB exacerbations or severe cough, short-term oral corticosteroids (e.g., prednisone 10–20 mg/d for 5–7 days) can provide rapid relief. Symptoms should be reassessed daily, and following improvement, ICS maintenance therapy should be initiated within 24 to 48 hours to minimize systemic risks, with gradual tapering of oral steroids in severe cases to prevent relapse. Novel peripheral antitussives such as levodropropizine act on peripheral cough receptors and have a favorable safety profile with moderate efficacy (e.g., 22% reduction in cough frequency). They are best used as adjunct therapy for refractory acute or CC, particularly along with etiology-specific treatments.^[57]^ Notably, studies have emphasized individualized treatment for CC, requiring comprehensive evaluation of etiology, inflammatory biomarkers (e.g., FeNO > 50 ppb, blood eosinophils >3%), drug response profiles, and adverse effects. These biomarkers can guide targeted selection and dose adjustment of anti-inflammatory therapies, enabling precision medicine approaches.^[97]^ Ongoing research into the molecular mechanisms of CC will likely yield more targeted, effective treatments.

##### 8.3.1.5. Innovation and application of non-pharmacological therapies

The comprehensive management of CC relies on pharmaceutical interventions and advancing non-pharmacological therapies – such as neuromodulatory devices and swallowing rehabilitation – which have demonstrated a 30% to 40% reduction in cough frequency when combined with pharmacotherapy. This dual approach has become an essential component of modern treatment strategies. Recent evidence supports the efficacy of targeted non-pharmacological interventions (e.g., neuromodulation for refractory cough, swallowing maneuvers for UACS) in specific types of CC, with response rates of 30% to 60%.^[98]^ For patients with RCC, these therapies can be used alone (achieving 45–60% response rates) or in combination with medication – such as pairing neuromodulation with ICS – which synergistically increases efficacy by 25% to 35% while broadening treatment options.^[99]^

##### 8.3.1.6. Breathing and cough control training

Cough suppression training, a behavioral intervention method based on neural plasticity, has shown increasing value in RCC management. It teaches patients to recognize pre-cough sensations and suppress cough impulses using specific techniques (e.g., paced breathing, voluntary cough suppression, swallowing maneuvers), achieving a 40% to 60% reduction in cough frequency in recent randomized trials.^[100]^ Clinical studies have shown that 6 to 8 weeks of structured behavioral training (e.g., cough suppression maneuvers) results in significant improvement (≥50% reduction in cough frequency) in 60% to 70% of patients with refractory UACS/GERC, with effects lasting 6 to 12 months.^[101]^ The training content typically includes paced abdominal breathing (respiratory reeducation), progressive muscle relaxation (relaxation techniques), cough suppression strategies (such as effortful swallowing and sipping water), and laryngeal protection techniques. This approach is particularly effective for patients with CHS and those with habitual cough behaviors due to CC, reducing cough frequency by 40% to 50% in clinical trials.^[102]^

Acoustic feedback therapy, an extension of respiratory training, is primarily used in patients whose cough is linked to vocal cord dysfunction. Real time auditory feedback improves vocal cord coordination and reduces spasmodic episodes.^[103]^ Speech therapists apply tailored voice therapy techniques and laryngeal muscle relaxation methods based on individual vocal cord movement assessments, aiming to reduce laryngeal irritation and cough reflex sensitivity by minimizing vocal fold collision.^[104]^ The 2025 International Aerosol Medicine Conference expert consensus recommends incorporating acoustic feedback therapy, particularly for patients with dysphonia or abnormal laryngeal sensation, into multidisciplinary CC management programs.^[105]^

##### 8.3.1.7. Environmental control and lifestyle intervention

Environmental control measures are a cornerstone of CC management. Expert consensus emphasizes avoiding exposure to smoke, dust, strong perfumes, and other volatile organic compounds, which can stimulate airway receptors and worsen cough.^[106]^ The use of high-efficiency air purifiers indoors significantly reduces airborne particulate matter and allergens, leading to >30% improvement in cough symptom scores, as supported by clinical studies. Maintaining indoor humidity between 40% and 60% is critical. Air that is too dry (<40%) can cause mucosal dryness and impair mucociliary function, while excessive humidity (60%) can promote fungal colonization (e.g., *Aspergillus* species); both conditions significantly aggravate cough symptoms.^[107]^

Dietary adjustments can further reduce cough severity (by 30–50%) in CC associated with GERC or UACS. This includes minimizing intake of spicy, acidic, or extreme temperature foods to limit acid reflux and direct airway irritation.^[108]^ Supplements such as vitamin C (500 mg/d), vitamin E (400 IU/d), and marine omega-3 fatty acids (eicosapentaenoic acid + docosahexaenoic acid, ≥1 g/d) may help reduce airway inflammation by neutralizing reactive oxygen species and modulating eicosanoid pathways, though current evidence remains limited to low-quality randomized controlled trial (GRADE C), with inconsistent effects on cough symptom scores. For GERC patients, additional measures include avoiding large meals, refraining from eating for 3 hours before bedtime, and elevating the head of the bed by 15 to 20 cm to reduce nocturnal acid reflux through gravitational effects, thereby minimizing esophageal acid exposure and lower esophageal sphincter relaxation episodes.

##### 8.3.1.8. Physical therapy and pulmonary rehabilitation

The pulmonary rehabilitation program, originally developed for patients with chronic obstructive pulmonary disease, has demonstrated significant benefits for CC patients, particularly those with airway mucus hypersecretion or mild airflow limitation.^[109]^ This program enhances cough effectiveness and exercise tolerance in CC patients, particularly those with airway mucus hypersecretion. This intervention benefits through postural drainage and positive expiratory pressure techniques, thereby improving airway clearance, and aerobic conditioning to optimize physical adaptation.^[110]^ Pulmonary rehabilitation integrates aerobic training to improve exercise capacity, cough training techniques (e.g., diaphragmatic coughing) to enhance cough efficacy, and behavioral strategies to minimize ineffective coughing episodes. An randomized controlled trial of 120 chronic bronchitis patients with CC found that a 12-week pulmonary rehabilitation program, including supervised exercise and airway clearance training, reduced Leicester Cough Questionnaire scores by 40% (from baseline 14 ± 3 to 8 ± 2) and significantly improved quality of life, as reflected in chronic obstructive pulmonary disease assessment test scores (from 20 to 12).^[111]^

Inspiratory muscle training, a core component of pulmonary rehabilitation, uses threshold inspiratory devices to provide resistive breathing exercises against incremental loads. This strengthens the diaphragm and improves endurance by increasing maximal inspiratory pressure and elevating neuromuscular efficiency, thus reducing breathlessness by 35% in CC patients with airway hyperresponsiveness.^[112]^ This training is especially beneficial for patients with CC-induced respiratory muscle fatigue, as it enhances cough peak flow and the mucus clearance efficiency. By strengthening expiratory muscles, intima-media thickness minimizes mucus stasis in lower airways, thereby preventing bacterial colonization and reducing the recurrence of lower respiratory tract infections. Patients receiving intima-media thickness experienced a 50% reduction in hospitalization for pneumonia compared with controls.^[113]^

##### 8.3.1.9. Psychological intervention and neural regulation techniques

CBT is increasingly recognized in CC management, particularly in patients with substantial anxiety or depression. CBT addresses maladaptive beliefs and reduces central sensitivity, significantly reducing cough frequency and improving quality of life in refractory cases, as shown in recent randomized trials.^[114]^ Psychological factors can heighten cough perception through glutamate-mediated central sensitization in the brainstem cough center. This can create a vicious cycle in which cough-induced anxiety further amplifies central hyperreactivity, as demonstrated by fMRI studies, which show that insular activation is associated with a 60% increase in cough reflex sensitivity.^[115]^ CBT helps patients identify and modify negative thought patterns and maladaptive coping strategies related to coughing, thereby reducing psychological distress and potentially decreasing both perceived and actual cough frequency.

This effect is thought to result from attenuation of glutamate-mediated central sensitization in the brainstem cough center, combined with cognitive restructuring to challenge catastrophic thoughts about coughing and behavioral strategies such as stimulus control training. This condition primarily affects school-age and adolescent children, presenting as a daytime, paroxysmal cough that disappears during focused activities or sleep. The cough is often high-pitched and brassy, resembling a honking sound, and is frequently associated with throat tension or emotional triggers.^[116]^

Neuromodulation techniques, including transcutaneous vagus nerve stimulation and other emerging methods, are under investigation. Early studies have suggested they reduce central cough sensitivity by modulating autonomic nervous system function.^[117]^ Although still experimental, these approaches show promise for highly refractory cases unresponsive to medication and behavioral therapies.

##### 8.3.1.10. Optimization of comprehensive intervention programs

The Guidelines for Chronic Cough Treatment emphasize the value of multidisciplinary collaboration, recommending coordinated management by specialists in respiratory medicine, otolaryngology, gastroenterology, speech-language pathology, and psychiatry for complex cases.^[118]^ This team-based approach ensures thorough evaluation of cough-related factors and the development of personalized intervention strategies. Clinical trials have demonstrated that patients enrolled in multidisciplinary cough programs achieve a 35% higher symptom improvement rate compared with conventional treatment, along with significantly better patient satisfaction scores.^[119]^ As the cornerstone of CC management, multimodal non-pharmacological therapies – including speech therapy and respiratory rehabilitation – tend to have a slower onset than pharmacological interventions. While exhibiting slower initial efficacy, these therapies offer superior safety profiles, minimal adverse effects, and have been shown to potentiate the drug. The 2024 treatment strategies place heightened emphasis on tailoring non-pharmacological interventions to patient-specific etiologies and cough phenotypes, systematically integrating these methods into individualized treatment plans.^[120]^ Ongoing research highlights their growing importance, with a projected expansion of their role in CC management.

##### 8.3.1.11. Management strategies for CC in elderly patients

Elderly patients with CC present unique challenges due to increased multimorbidity and polypharmacy. In addition to common etiological factors, drug side effects (e.g., ACEI antihypertensive drugs), subclinical microaspiration linked to age-related swallowing impairment, and systemic conditions such as chronic heart failure are more prevalent in this group.^[121]^

In elderly patients with CVA, ICS are effective, but require careful monitoring for risks such as osteoporosis and oral candidiasis. Adding leukotriene receptor antagonists (e.g., montelukast) can reduce ICS dosage, lowering side effects and improving treatment outcomes, particularly in those with comorbid allergic rhinitis.^[122]^

In GERC, standard treatment often involves prolonged PPI therapy for up to 12 weeks for elderly patients, often in combination with gastrointestinal motility agents (e.g., domperidone). However, novel potassium-competitive acid blockers such as vonoprazan provide stronger and longer-lasting acid suppression, potentially shortening treatment duration and improving symptom control in elderly patients.^[123]^

ACEI medication-induced cough occurs in 5% to 35% of elderly users. The 2022 guideline recommends switching to angiotensin receptor blockers or angiotensin receptor-neprilysin inhibitors in patients intolerant to ACEIs.^[2]^

CC assessment in elderly patients demands careful assessment of extrapulmonary symptoms and underlying systemic conditions. Because atypical symptom presentation is common in older adults, a comprehensive diagnostic approach is usually required. For example, chest CT can reveal occult pulmonary fibrosis or early tumors, while swallowing function evaluations can detect silent aspiration. The impact of drug interactions and changes in liver/kidney function on drug metabolism must be considered during treatment. The principle of “low-dose initiation and gradual titration” should be adopted, with simultaneous close monitoring of adverse reactions.

##### 8.3.1.12. Cough management in patients with chronic diseases

The 2022 GOLD guidelines highlight CC in chronic obstructive pulmonary disease, where cough results from multiple interacting factors, including airway inflammation, mucus hypersecretion, and structural airway remodeling. The guidelines recommend administering a combination of dual bronchodilators (salmeterol and bambuterol) as first-line treatment for CC in chronic obstructive pulmonary disease, significantly improving cough symptoms.^[124]^ In eosinophilic chronic obstructive pulmonary disease, ICS are recommended, but pneumonia risk must be considered. Cough in chronic heart failure is often overlooked but may result from pulmonary congestion, ACEI medications, or comorbid airway diseases. Optimizing heart failure management, including diuretic adjustment, can reduce cardiogenic cough; however, other causes should be excluded to ensure appropriate targeted treatment strategies.^[11]^ In lung cancer survivors, CC can severely impair quality of life. Neuromodulators (e.g., gabapentin) and speech therapy interventions have shown benefit in these patients.^[125]^

##### 8.3.1.13. Gender differences and individualized treatment

Research has increasingly focused on gender differences in CC. Women – particularly postmenopausal women – show higher cough sensitivity and a greater incidence of NAEB than men.^[126]^ Hormonal influences, heightened cough reflex sensitivity, and psychosocial factors likely contribute to this gender disparity. CC management in women should prioritize cough suppression training and psychological support, especially for those with pronounced sensitivity. Special population management must balance efficacy and safety, considering age-related pathophysiological changes and comorbidities. Guidelines advocate a multidisciplinary, individualized approach, selecting diagnostic and treatment strategies according to population-specific characteristics to optimize relief and minimize risks. This approach aims to optimize symptom relief while mitigating treatment-related risks.^[121]^ As the understanding of CC in special populations deepens, diagnostic and treatment strategies are expected to evolve into more precise and patient-centered approaches.

In summary, advancements and applications in diagnostic and treatment technologies include promoting long-term cough frequency monitoring (>24 hours) to minimize subjective scoring errors; AI-assisted analysis of symptoms and investigations to improve diagnosis accuracy (e.g., for rare causes such as retrosternal goiter and giant cell arteritis); deep diaphragmatic breathing training, improving GERC remission symptoms (complete remission rate: 27% vs 7% in controlled trials); and posterior nasal nerve radiofrequency thermocoagulation for reducing UACS.^[127,128]^ Emerging drugs in clinical trials include HS-10383 and BLU-5937, along with receptor antagonists targeting P2X3 (camlipixant, with the lowest dysgeusia risk), TRPV4 (GSK2798745, for hypersensitive cough), histamine H4 (ADX-629, with potential anti-inflammatory and antitussive dual effects), and TRPM8 (AX-8, addressing temperature-triggered cough) advancing to phase II/III clinical trials.^[129–132]^ Neuroregulatory research into brainstem cough center circuits may lead to neuroablation and neuromodulation therapies.^[133–135]^

## 9. Future research directions and clinical prospects

### 9.1. Future research directions

Research on CC has progressed rapidly in 2025. With growing insights into disease mechanisms and the emergence of novel technologies, further breakthroughs are anticipated in the coming years. Building on current clinical trial findings and expert consensus, future research will likely prioritize the following areas.

#### 9.1.1. Cough mechanism and biomarker research

The neurobiological mechanisms underlying cough reflex sensitivity will remain a central focus of future research. RCC may represent an idiopathic neurosensory disorder characterized by peripheral and central sensitization. Key mechanisms underlying RCC involve upregulated expression of cough receptors (e.g., TRPV1 and TRPA1 ion channels), increased excitability of vagal afferent pathways, impaired inhibitory function within the brainstem cough center, and dysregulated cortical modulation of cough.^[136]^ Future research will aim to clarify how these multilevel abnormalities interact and to identify molecular biomarkers for diagnosis and therapeutic targeting.

Biomarker studies remain highly active. Traditional markers (e.g., induced sputum eosinophil count and fractional exhaled FeNO) are now complemented by emerging candidates. Novel blood-based biomarkers, including eosinophil cationic protein, eosinophil peroxidase, and eosinophil-derived neurotoxin, are under evaluation for improving disease stratification and treatment monitoring.^[137–139]^ Exhaled volatile organic compounds analysis offers a fully noninvasive means of differentiating CC subtypes through disease-specific metabolic signatures. Preliminary studies have reported sensitivity and specificity exceeding 80%, suggesting that VOC profiling could replace or supplement invasive tests in precise CC phenotyping.^[84]^

Genetic and epigenetic research is also shedding light on individual susceptibility to CC. Genome-wide association studies have identified multiple genetic loci linked to cough sensitivity, including P2X3 receptor polymorphisms – specific variants of which predict response to gefapixant treatment.^[45,140]^ Epigenetic modifications, such as DNA methylation, may mediate the effects of environmental exposures on CC by modulating gene expression. These mechanisms offer novel targets for developing preventive or therapeutic interventions, potentially paving the way for precision medicine approaches in cough management.

#### 9.1.2. Targeted drug development and clinical trials

The rational design and optimization of novel P2X3 receptor antagonists remain a key focus in drug development for CC, driven by their potential to modulate cough sensitivity.^[141,142]^ Although the first-generation P2X3 antagonist gefapixant has been approved for clinical use, it causes taste disturbances in up to 30% of patients – significantly limiting its widespread adoption in CC management.^[143,144]^ Second-generation P2X3 antagonists, such as camipixant, are expected to enter phase III trials in 2025. These agents exhibit greater selectivity for specific P2X3 receptor subtypes, significantly reducing taste-related adverse effects while sustaining efficacy. Within the next 5 years, 2 to 3 new P2X3 inhibitors may receive market approval, expanding treatment options for RCC patients.

Neurokinin-1 receptor antagonists also show promise by blocking substance P, a key neuropeptide in nociceptive signaling. Elevated NK-1 receptor expression in airway sensory nerves of CC patients supports their potential therapeutic role.^[145]^ Early-phase trials indicate that certain neurokinin-1 and P2X3 receptor antagonists, currently in preclinical and phase 1/2 trials, may benefit CC patients with comorbid laryngeal hypersensitivity and anxiety/depression, minimizing central nervous system side effects.

Targeted biologics are being investigated for specific CC phenotypes, particularly those linked to type 2 inflammation. Agents targeting the IL-4/IL-13 axis, such as dupilumab, are in phase II/III trials for patients with NAEB and comorbid asthma.^[146]^ Monoclonal antibodies against thymic stromal lymphopoietin have shown efficacy in reducing cough frequency and sputum eosinophil counts in patients with severe NAEB.^[147]^ Although these high-cost therapies show promise, future research will prioritize identifying biomarkers or phenotypic features for predicting CC subgroups that are most likely to respond.

Advancements in inhalation drug delivery systems are enhancing drug efficacy. Novel smart nebulizers can analyze real time breathing patterns, dynamically adjusting aerosol particle size and flow during inspiratory/expiratory phases, thereby enhancing pulmonary deposition by up to 40% compared with conventional devices.^[148]^ Sustained-release nanoparticle formulations for targeted pulmonary delivery have shown the ability to maintain anti-type 2 inflammatory effects for up to 3 weeks after a single inhalation in preclinical studies.^[149]^ Such innovations could improve patient adherence challenges while complementing the development of novel biologic therapy, offering a transformative approach to CC management.

#### 9.1.3. Precision medicine and artificial intelligence applications

Molecular classification and precision medicine are emerging as a future paradigm in CC management. Utilizing multi-omics approaches (transcriptomics, proteomics, metabolomics), the present research aims to identify distinct CC subtypes – such as “Th2-high” and “Th2-low” NAEB – and neural sensitivity phenotypes to identify disease mechanisms and inform targeted therapies.^[74]^ This molecular classification system will aid clinicians in personalizing treatment strategies: for example, anti-IL-5 biologics target Th2-high subtypes by inhibiting eosinophilic inflammation, while P2X3 receptor antagonists address neural sensitivity phenotypes, such as CHS, potentially reducing cough frequency and improving the quality of life of patients.

AI-powered cough monitoring has significantly advanced, leveraging smartphone-based deep learning algorithms to extract acoustic biomarkers from cough sounds. This allows quantifying cough frequency objectively and detecting CC syndromes early.^[150]^ According to preliminary studies, its accuracy in distinguishing CVA, UACS, and GERC reaches 75% to 85%.^[151]^ Such tools may enable effective screening of CC in primary care and telemedicine settings.

Digital therapeutics, an emerging field integrating behavioral interventions, respiratory biofeedback, and remote health monitoring, seem promising through digital cough rehabilitation programs. After being validated in clinical trials, these programs leverage smartphones to deliver cough profile-based personalized training plans to patients, offer real time feedback on breathing mechanics, and seamlessly share physiological data with healthcare teams – aiding remote management and optimizing treatment adherence.^[152]^ This approach can significantly expand access to non-pharmacological therapies, particularly for patients with geographic or mobility barriers to engage in evidence-based cough rehabilitation programs from home.

#### 9.1.4. Clinical practice and public health implications

The 2025 Global Initiative on CC guidelines update is set to integrate Grade A evidence from landmark trials, emphasizing biomarker-based diagnostics and digital therapeutics within treatment algorithms.^[153]^ Anticipated updates in the 2026 international guidelines for CC may include several crucial adjustments: standardizing the sputum eosinophil threshold to 2.5% in diagnosing NAEB; adding P2X3 antagonists as first-line treatment for RCC; and clarifying the use of biologic agents in specific CC phenotypes.

Although long-term data remain limited, cohort studies have suggested that untreated NAEB patients carry a 9% to 16% risk of progressing to asthma or fixed airway obstruction over 5 to 10 years. Future large-scale longitudinal studies are warranted to clarify the natural history of CC subtypes and determine how targeted early interventions can influence disease progression. Such evidence will be pivotal for shaping public health strategies, including population screening programs and treatment algorithm prioritization.

Improving education and awareness among both patients and healthcare providers is essential for optimizing CC management. Evidence shows that patients often consult an average of 3.5 physicians before receiving an accurate diagnosis, with delays spanning months to years – underscoring the need for targeted education.^[80]^ Future general practitioner training should prioritize evidence-based CC management pathways, including guideline-recommended therapies such as PPIs for CVA. Concurrently, public health campaigns should also address misconceptions about cough self-management – for example, educating communities that a cough persisting more than 3 weeks warrants specialist evaluation rather than over-the-counter antibiotics.

### 9.2. Prospect

The future of CC treatment is moving decisively toward precision medicine, shifting away from purely symptomatic management. Over the next 5 to 10 years, several shifts are anticipated: for eosinophilic cough subtypes, standardized biomarker panels (comprising blood eosinophil count, fractional exhaled FeNO, and induced sputum eosinophilic cationic protein) are expected to replace bronchoscopy in many diagnostic workflows. The 2023 American Thoracic Society guidelines already report 85% diagnostic accuracy without invasive sampling. Targeted therapies for refractory CC will increasingly focus on disease-specific pathways: monoclonal antibodies against IL-5 (e.g., benralizumab for eosinophilic CC) and P2X3 antagonists for neuropathic cough are advancing through phase III trials, and thymic stromal lymphopoietin inhibitors seem promising for atopic phenotypes. This precision approach enables personalized management, as recommended by the 2022 European Respiratory Society guidelines; multimodal interventions for refractory CC will increasingly combine drug therapy (e.g., biologics for eosinophilic phenotypes) and non-pharmacological strategies, including digital tools for cough monitoring and behavioral therapies such as speech pathology-guided cough suppression training randomized controlled trials have shown 30% symptom reduction compared with monotherapy (2023 Cochrane review). This approach is also recommended by European Respiratory Society/American College of Clinical Pharmacy guidelines; holistic management: Expanding beyond acute symptom control to comprehensive management, including acute phase treatment, maintenance therapy, relapse prevention, and quality of life improvement; and systematic research: Prospective large-scale registry and real-world evidence studies will generate robust longitudinal data on efficacy, safety, and health economics, facilitating evidence-based clinical decision-making for personalized medicine and regulatory approvals.

With the translation and integration of novel therapies, real-world evidence, and mechanistic insights, CC is poised to transition from an often underdiagnosed symptom cluster to a defined disease entity, characterized by standardized diagnostics, targeted treatments, and measurable quality-of-life outcomes. Patient-centered care models will empower individuals to become active participants in their management through digital tools and shared decision-making. This evolution aims for precise phenotyping, personalized interventions, and proactive risk mitigation, thereby improving long-term outcomes and restoring both quality of life and societal productivity. The continuous emergence and clinical translation of novel concepts, therapies, and digital health technologies are reshaping CC management, marking a major shift in diagnosis and treatment. Despite the challenges remaining in implementation and equity, the future of CC management appears more promising than ever, offering renewed hope to patients with longstanding disease.

## 10. Summary

The most common causes of CC are CVA, GERC, NAEB, and UACS, with incidence increasing globally, particularly in regions such as North America and Western Europe. Key cough neuroreflex mechanisms involve activation of extrapulmonary C-fibers, pulmonary mechanoreceptors (such as RARs and SARs), neurogenic inflammation, and TRPV1 receptors. CC presents with diverse and complex phenotypes, necessitating a comprehensive yet targeted diagnostic evaluation that integrates neural mechanisms, environmental exposures, and individualized assessment. While advances have been made in targeted drug and nondrug interventions, challenges persist in achieving standardized diagnosis and treatment – especially in primary care – and in elucidating mechanisms underlying RCC analysis. Addressing these gaps will require deeper investigation into pathophysiological mechanisms (such as neurogenic inflammation and abnormal receptor signaling) and the development of targeted agents (e.g., TRPV1 antagonists, neuropeptide inhibitors), aiming to unlock new solutions for clinical management. Strengthening primary healthcare capacity to identify CC subtypes and adopt targeted therapies is essential for improving diagnostic accuracy and treatment efficacy at the community level. Transitioning from symptom-based treatment to disease eradication requires a trifecta of scientific breakthroughs (discovery of new molecular targets), technological integration (AI-enabled monitoring), and systemic reform (primary care–specialty care collaboration).

## Acknowledgments

We would like to thank Prof Liangan Chen, Department of Respiration in People’s Liberation Army General Hospital for our guidance.

## Author contributions

**Conceptualization:** Xiao-ji Zhu, Xiaoxuan Hu, Keli Zhang.

**Data curation:** Keli Zhang.

**Formal analysis:** Tao Liu.

**Funding acquisition:** Xiao-ji Zhu.

**Investigation:** Xiao-ji Zhu, Xiaoxuan Hu, Tao Liu.

**Project administration:** Xiao-ji Zhu.

**Writing – review & editing:** Xiao-ji Zhu.

**Writing – original draft:** Xiaoxuan Hu, Keli Zhang.
